# Chest pain after elective percutaneous coronary intervention as trigger of takotsubo syndrome—a case report

**DOI:** 10.1093/ehjcr/ytae694

**Published:** 2024-12-26

**Authors:** Vincenzo Castiglione, Chiara Arzilli, Marco Ciardetti, Michele Emdin, Michele Coceani

**Affiliations:** Fondazione Toscana Gabriele Monasterio, Via Giuseppe Moruzzi 1, 56124 Pisa, Italy; Health Science Interdisciplinary Center, Scuola Superiore Sant’Anna, Piazza Martiri della Libertà 33, 56127 Pisa, Italy; Fondazione Toscana Gabriele Monasterio, Via Giuseppe Moruzzi 1, 56124 Pisa, Italy; Fondazione Toscana Gabriele Monasterio, Via Giuseppe Moruzzi 1, 56124 Pisa, Italy; Fondazione Toscana Gabriele Monasterio, Via Giuseppe Moruzzi 1, 56124 Pisa, Italy; Health Science Interdisciplinary Center, Scuola Superiore Sant’Anna, Piazza Martiri della Libertà 33, 56127 Pisa, Italy; Fondazione Toscana Gabriele Monasterio, Via Giuseppe Moruzzi 1, 56124 Pisa, Italy

**Keywords:** Takotsubo syndrome, Percutaneous coronary intervention, Angina, Cardiac arrest, Case report

## Abstract

**Background:**

Takotsubo syndrome (TTS) is characterized by transient left ventricular dysfunction, often triggered by emotional or physical stress. It usually presents with clinical features similar to acute coronary syndrome, making its occurrence following elective percutaneous coronary intervention (PCI) challenging to diagnose and treat.

**Case summary:**

A 67-year-old man with ischaemic heart disease and recurrent angina underwent elective PCI of the right coronary artery. The procedure, although technically challenging, was completed without immediate complications. However, shortly after the intervention, the patient experienced acute chest pain, initially thought to be due to subocclusion of a postero-lateral branch, which was treated with balloon angioplasty. Despite this intervention, the patient developed severe ventricular arrhythmias and exhibited dynamic electrocardiographic changes and echocardiographic features consistent with TTS. Cardiac magnetic resonance (CMR) imaging confirmed the diagnosis, revealing classic apical ballooning and left ventricular dysfunction. With comprehensive medical management and haemodynamic support, the patient gradually recovered. He was discharged after stabilization, with follow-up showing complete resolution of the left ventricular dysfunction.

**Discussion:**

This case highlights the importance of recognizing TTS as a potential complication following PCI, particularly in patients with a heightened stress response. It emphasizes the need for increased awareness and the use of advanced diagnostic tools, such as CMR imaging, to accurately identify TTS. Early diagnosis and appropriate management are crucial for improving outcomes, especially in complex PCI cases where TTS can mimic more common coronary complications.

**Learning points**

- Explore preventive and management strategies to mitigate the risk of takotsubo syndrome (TTS) in patients undergoing percutaneous coronary intervention, emphasizing chest pain control, and recognizing procedural complexities.
- Understand the role of cardiac magnetic resonance in confirming TTS diagnosis and its impact on clinical management.

## Introduction

Takotsubo syndrome (TTS) is characterized by sudden, usually reversible left ventricular systolic dysfunction that often mimics acute coronary syndromes.^1^ Typically triggered by severe stress—physical or emotional—such as major illness, surgery, or emotional trauma, TTS may also arise perioperatively, during or after procedures like anaesthesia induction or surgery.^2^ The condition often presents with acute chest pain, electrocardiographic (ECG) changes, and sudden left ventricular systolic dysfunction, though most patients recover within weeks. Despite this, TTS can lead to serious complications, including ventricular arrhythmias due to adrenergic stress and prolonged QT intervals.^1^ Differentiating TTS from acute coronary syndrome is challenging but crucial for effective management.^3^

## Summary figure

**Figure:**
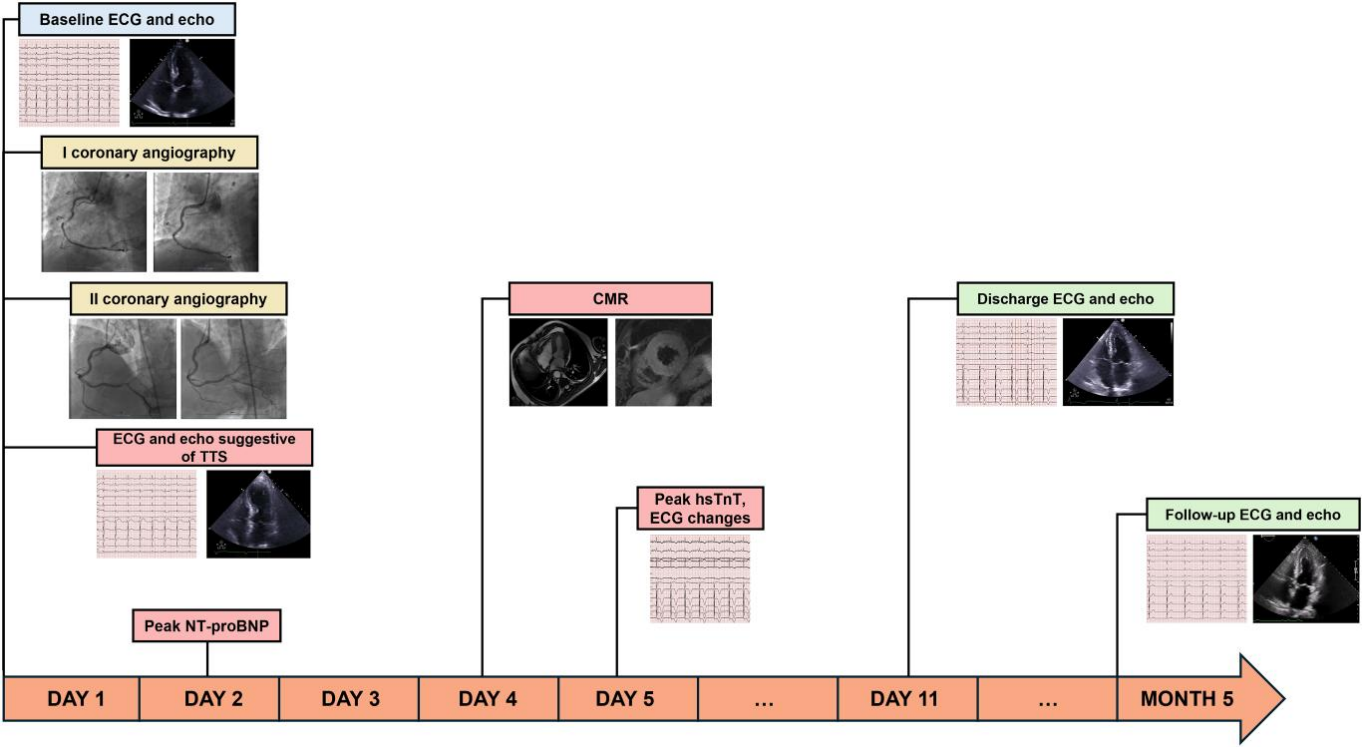


## Case presentation

A 67-year-old man was admitted to our hospital due to worsening angina (Grade III Canadian Cardiovascular Society). He had a significant cardiovascular risk profile, including a family history of ischaemic heart disease, active smoking (35 pack-years), hypertension, dyslipidaemia, and stage three chronic kidney disease. He had already undergone percutaneous coronary interventions (PCI) of the circumflex and right coronary arteries (RCA) in 2008, 2017, and 2022 for non-ST-elevation myocardial infarctions.

**Figure 1:**
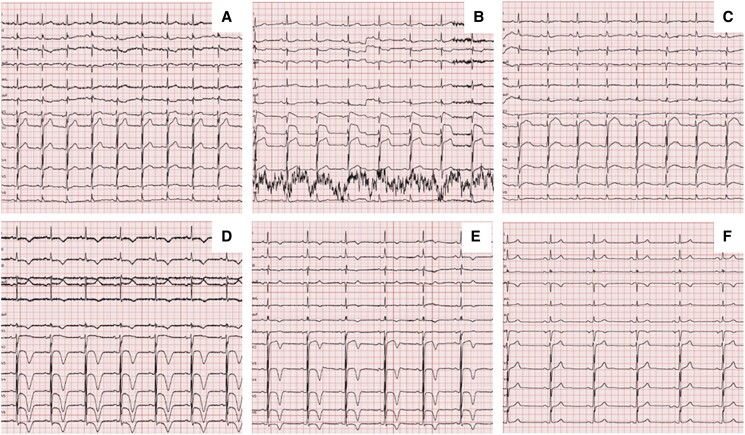
Electrocardiographic dynamic changes. Progressive development of electrocardiographic alterations typical of takotsubo syndrome, including ST-segment elevation, QTc prolongation, and T-wave inversion.

A recent ergometric stress test had induced angina at a 50-W workload without significant ECG changes. Physical examination was unremarkable, albeit the patient appeared anxious. Electrocardiographic showed normal sinus rhythm with Q waves in lead III and biphasic T waves in lead III and aVF leads (*Figure 1A*), whereas echocardiography revealed a 65% left ventricular ejection fraction (LVEF; Supplementary material online, *Videos S1* and *S2*).

**Figure 2:**
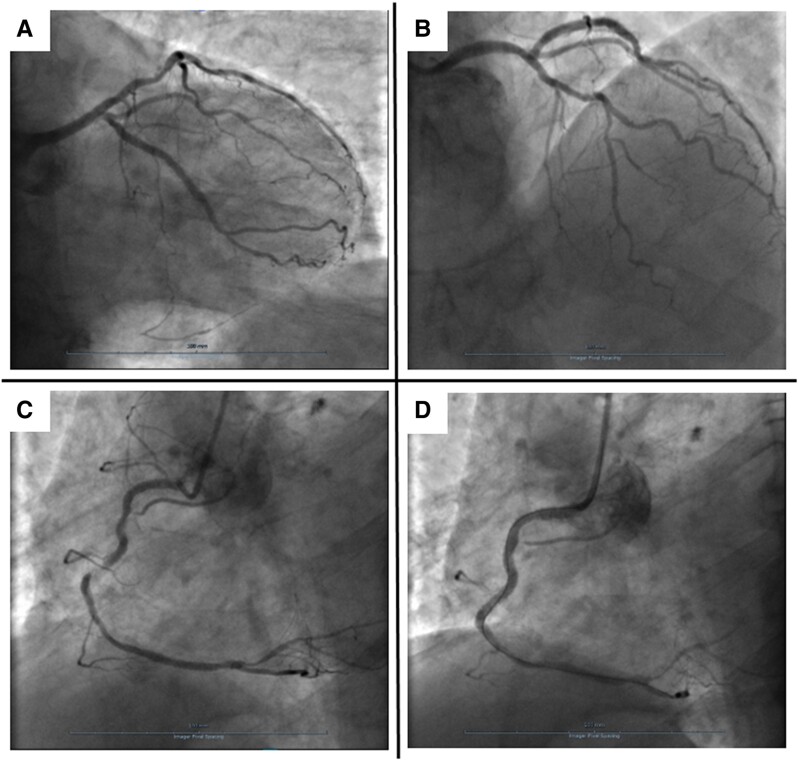
Coronary angiography. Coronary angiography views of the left coronary artery (*A*, *B*) and right coronary artery before (*C*) and after (*D*) the first percutaneous coronary angioplasty.

**Figure 3:**
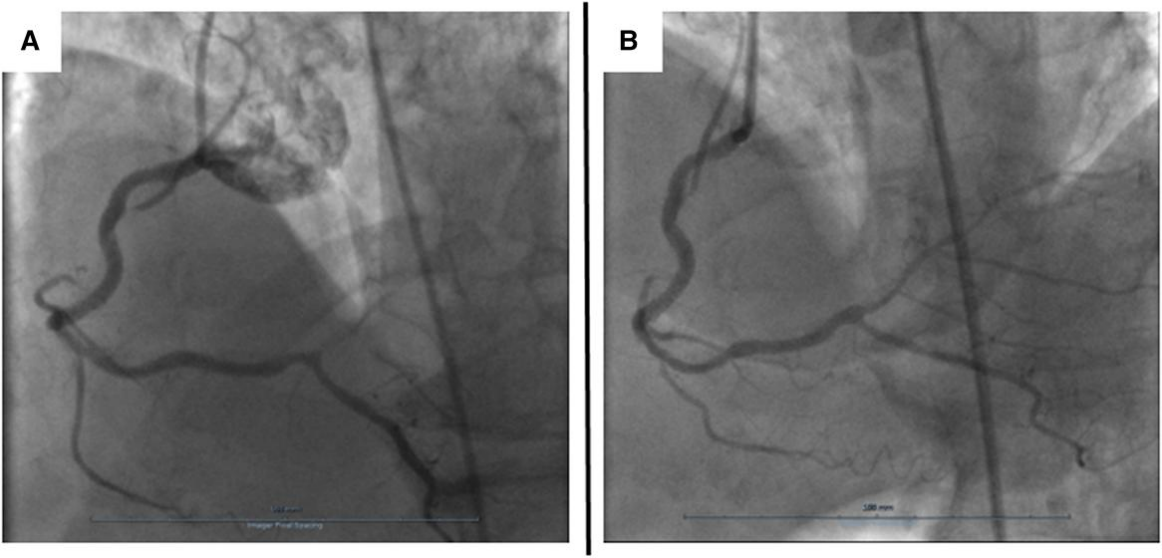
Coronary angiography. Coronary angiography views of the right coronary artery before (*A*) and after (*B*) the second percutaneous coronary angioplasty.

**Figure 4:**
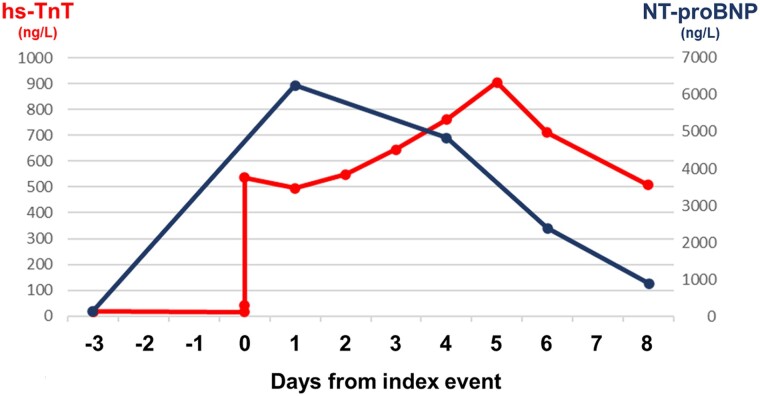
Cardiac biomarkers levels. Dynamic changes in high-sensitivity troponin T and N-terminal pro-B-type natriuretic peptide levels during hospitalization. Cardiac biomarkers levels from a previous blood sample performed before hospitalization are also reported.

**Figure 5:**
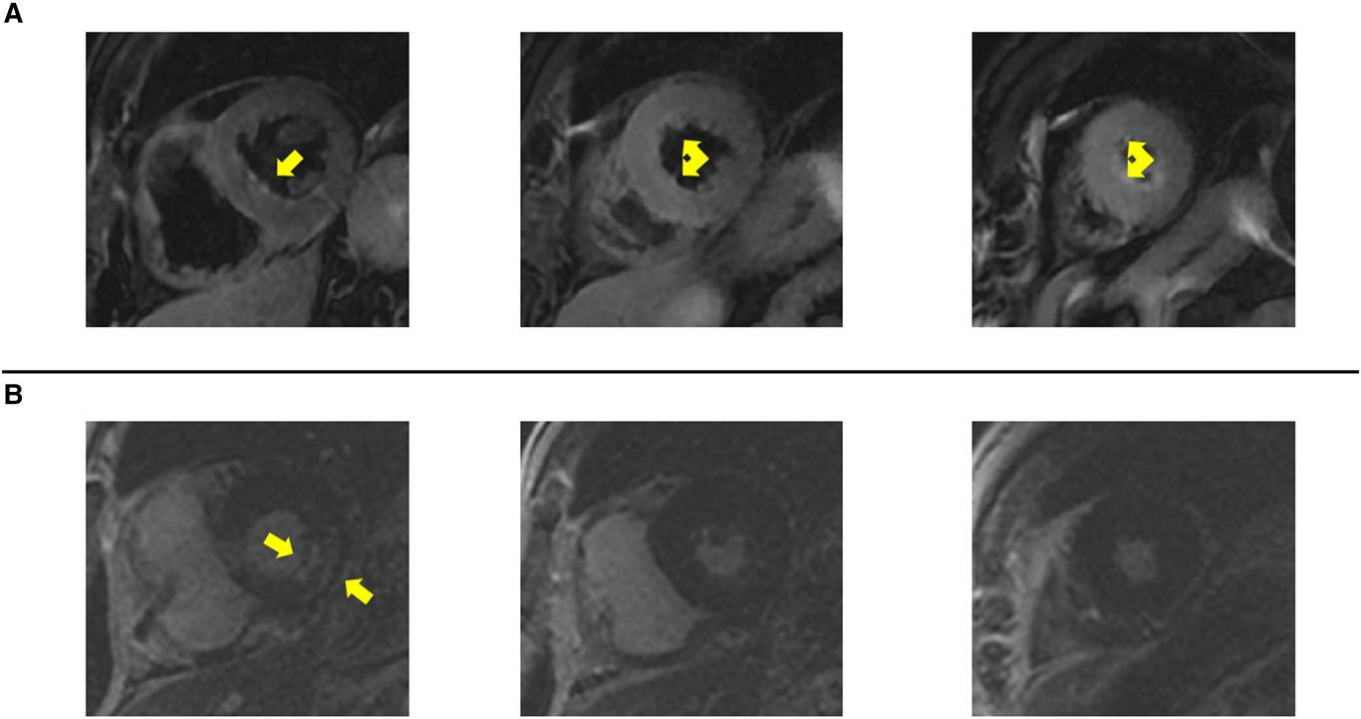
Cardiac magnetic resonance T2-weighted short-tau inversion recovery sequences (short-axis views; *A*) show diffuse transmural oedema (arrows) involving the apical and mid-segments of the septum, anterior, and inferior walls, as well as subendocardial oedema of the basal infero-lateral wall. T1-weighted gradient echo-inversion recovery sequences (short-axis views; *B*) show late gadolinium enhancement (arrows) limited to the basal infero-lateral wall.

Coronary angiography showed patency of previous stents and non-significant atherosclerosis in the left anterior descending (LAD) artery (*Figure 2A* and *B*; Supplementary material online, *Videos S3* and *S4*). Conversely, the RCA had a significant stenosis at the ostium and a subocclusive in-stent restenosis in the mid-segment (*Figure 2C*; Supplementary material online, *Video S5*), which were challenging to treat due to heavy calcifications and vessel tortuosity. After successfully placing three drug-eluting stents in the RCA (*Figure 2D*; Supplementary material online, *Video S6*), the patient was transferred to the intensive care unit (ICU) for observation. Once there, he started complaining of worsening chest pain, and a 12-lead ECG showed ST-segment elevation in V1-V3, suggesting LAD territory ischaemia (*Figure 1B*). Cardiac contractility appeared normal on echocardiography (see Supplementary material online, *Video S7*). Given the evolving clinical scenario, a second coronary angiogram was performed, which demonstrated patency of all stents, but identified subocclusion of the RCA postero-lateral branch (*Figure 3A*; Supplementary material online, *Video S8*), which was addressed with successful balloon angioplasty (*Figure 3B*; Supplementary material online, *Video S9*), with complete relief of chest pain. Nevertheless, shortly after returning to the ICU, the patient experienced recurrent episodes of sustained polymorphic ventricular tachycardia and ventricular fibrillation, requiring seven external direct current shocks, and cardiopulmonary resuscitation for 15 min. Post-resuscitation, a 12-lead ECG showed sinus rhythm, persistent ST-segment elevation in the precordial leads, and new-onset QT interval prolongation (500 ms; *Figure 1C*). Echocardiography revealed akinesia involving all LV apical segments (see Supplementary material online, *Video S10*), and, within a few minutes, also all mid-segments, accompanied by hyperkinesia of the basal segments and severely impaired global LV systolic function (LVEF 35%), leading to the suspicion of TTS (see Supplementary material online, *Video S11*). Due to the persistence of ECG changes and arterial hypotension, the patient underwent a third coronary angiogram, which did not reveal any significant new coronary abnormalities, as well as an intra-aortic balloon pump placement. Interventions in the ICU included the administration of intravenous noradrenaline, magnesium sulfate, and lidocaine. The patient gradually improved over 72 h, allowing removal of the intra-aortic balloon pump and cessation of intravenous therapies. N-terminal pro-B-type natriuretic peptide peaked at 6229 ng/L (normal range <125 ng/L) after 24 h, while high-sensitivity troponin T peaked at 906 ng/L (normal range <14 ng/L) after 5 days (*Figure 4*). The patient was prescribed dual antiplatelet therapy (aspirin and ticagrelor), atorvastatin/ezetimibe 10/10 mg combination, as well as guideline-directed anti-remodelling medications: metoprolol 50 mg b.i.d, sacubitril/valsartan 24/26 mg b.i.d., eplerenone 50 mg, and empagliflozin 10 mg. Cardiac magnetic resonance (CMR) imaging performed three days after the cardiac arrest confirmed the diagnosis of TTS, while demonstrating a partial improvement in LV systolic dysfunction (LVEF 45%) and, notably, diffuse myocardial oedema without evidence of delayed enhancement in the LAD territory (*Figure 5*; Supplementary material online, *Video S12*). Profound precordial negative T waves were evident after 4 days (*Figure 1D* and *E*). Echocardiography revealed a trend towards progressive normalization of LV systolic function (LVEF 55% at discharge, after 10 days), which began from the mid-segments (see Supplementary material online, *Video S13*) and extended towards the apex (see Supplementary material online, *Video S14*).

At the follow-up visit in January 2024, 5 months post-event, the patient was completely asymptomatic, with resolved T-wave abnormalities (*Figure 1F*) and normal LV function (see Supplementary material online, *Video S15*).

## Discussion

This case illustrates the occurrence of TTS following elective PCI, emphasizing the diagnostic and management challenges posed by this complication. The initial presentation with acute chest pain, ST-segment elevation, and ventricular arrhythmias immediately post-PCI underscores the need for a systematic evaluation of coronary causes (e.g. stent thrombosis, in-stent restenosis, and distal embolization) as the primary differential diagnosis in such scenarios. In this case, the early post-PCI chest pain was attributed to subocclusion of the postero-lateral branch, which was successfully treated with balloon angioplasty. However, the subsequent recurrence of chest pain, severe ventricular arrhythmias, and dynamic echocardiographic changes necessitated a broader diagnostic approach, pointing towards a non-coronary aetiology. This case highlights the critical importance of ruling out ischaemic complications and revascularization-related issues before considering alternative diagnoses like TTS. Such a stepwise approach ensures timely identification of life-threatening conditions while avoiding premature conclusions.

Takotsubo syndrome is typically precipitated by intense physical or emotional stress, leading to a catecholamine surge.^1^ Takotsubo syndrome can also occur perioperatively, after anaesthesia, cardiac surgery, or invasive medical procedures, such as electrophysiological procedures, percutaneous valve repair, and replacement.^2^ This is often due to a combination of physical and emotional stress, exacerbated by anaesthetics and vasopressors increasing blood catecholamines.^4^ In this case, TTS developed following elective PCI, a rarely reported trigger.^5–8^ Previous similar cases linked TTS to inadvertent norepinephrine administration,^8^ or inadequate management of perioperative anxiety and post-revascularization angina.^5–7^ These reports, along with ours, emphasize the need for TTS prevention during elective PCI. Considering the role of adrenergic activation in TTS pathophysiology, it is imperative to guarantee effective management of periprocedural chest pain, especially when a technical complexity, as seen in our case, is anticipated.

Distinguishing TTS from acute myocardial infarction can be challenging due to similar ECG, biomarker, haemodynamic, and echocardiographic abnormalities.^3^ Takotsubo syndrome typically presents with higher natriuretic peptide levels, reflecting increased filling pressures, and lower troponin levels,^9^ as observed here. However, these differences alone are often insufficient for diagnosis, necessitating multimodal assessment. In this case, CMR was crucial in confirming TTS by revealing myocardial oedema without fibrosis, a hallmark of TTS.^1^ It also helped downplay the role of the occlusion of the postero-lateral branch, which may have caused the chest pain triggering TTS but was not the cause of the ECG and echocardiographic changes. Serial CMR testing could provide further insights into myocardial recovery over time. A previous study of TTS patients showed that myocardial alterations observed on CMR typically resolve by 180 days.^10^ Therefore, follow-up CMR, ideally around 6 months after the initial TTS diagnosis, may be beneficial to monitor recovery.

An unique aspect of this case was the time course of TTS-related ventricular dysfunction: initial LV dysfunction was noted in the apex, later involving mid-segments, with recovery starting in the mid-segments and extending to the apex.^11^ In this case, we chose to implement the four pillars of heart failure therapy in the acute phase to promote the recovery of cardiac function. Nevertheless, there is currently no consensus on the indication or duration of guideline-directed medical therapy after TTS, with mixed evidence regarding the use of beta-blockers and angiotensin-converting enzyme inhibitors/angiotensin II receptor blockers.^12^ The patient was continued on a beta-blocker for its anti-ischaemic properties, given the history of coronary syndrome, while the mineralocorticoid receptor antagonists and the sodium-glucose co-transporter 2 inhibitors were maintained due to their benefits in chronic kidney disease.^13^ Sacubitril/valsartan was also continued based on preclinical evidence supporting its use in TTS.^14^

Finally, this case underscores the potential for electrical instability in TTS, as evidenced by the occurrence of an arrhythmic storm, likely due to adrenergic activation triggered by sustained intense chest pain, and possibly favoured by QTc prolongation (reaching a maximum of 570 ms during the hospitalization, *Figure 1D*), which is known as one of the strongest predictors of ventricular arrhythmias in TTS.^15^ Indeed, a QTc value ≥460 ms has been linked to a higher risk for in-hospital arrhythmic complications in patients with TTS.^16^ Therefore, close ECG monitoring is warranted in patients with the suspicion of TTS.^12^ In this case, we decided not to implant a cardioverter-defibrillator for secondary prevention, as ventricular arrhythmias in TTS are usually associated with ‘reversible’ triggers of electrical instability, and cardioverter-defibrillator implantation has not been shown to provide benefit.^17^

In summary, this case describes the occurrence of TTS following elective PCI, possibly elicited by periprocedural stress. Close monitoring and multimodal assessment, including cardiac CMR, are vital for accurate diagnosis. The patient responded well to comprehensive management and was discharged with normal LV systolic function, underscoring the importance of early recognition and appropriate care.

## Supplementary Material

ytae694_Supplementary_Data

## Data Availability

The data underlying this article will be shared on reasonable request to the corresponding author.

## Lead author biography

I am a cardiologist at Fondazione Toscana Gabriele Monasterio (Pisa) and a PhD student in Translational Medicine at Sant’Anna School of Advanced Studies (Pisa). I graduated in Medicine from the University of Pisa in 2017 and was also an Honour Course student at Sant’Anna School of Advanced Studies, from which I graduated in 2018. My research focuses mainly on heart failure and cardiomyopathies.

## Notes

**Consent:** The authors confirm that consent for the submission and publication of this case report, including images, videos, and associated text, was obtained from the patient in accordance with COPE guidance.

**Funding:** None declared.
